# A Review of Pharmacologic and Non-Pharmacologic Therapies in the Management of Irritable Bowel Syndrome: Current Recommendations and Evidence

**DOI:** 10.3390/jcm13226948

**Published:** 2024-11-18

**Authors:** Anthony J. Papale, Robert Flattau, Nandan Vithlani, Deepti Mahajan, Sandeep Nadella

**Affiliations:** 1 Department of Medicine, North Shore University Hospital/Zucker School of Medicine at Hofstra University, Manhasset, NY 11030, USA; dmahajan2@northwell.edu; 2 Zucker School of Medicine at Hofstra University, Hempstead, NY 11549, USA; rflattau@northwell.edu (R.F.); nvithlani@northwell.edu (N.V.); 3 Division of Gastroenterology, Department of Medicine, Northwell Health, New Hyde Park, NY 11040, USA; snadella@northwell.edu; 4 Cold Spring Harbor Laboratory, Cold Spring Harbor, NY 11724, USA

**Keywords:** irritable bowel syndrome, IBS, Rome IV, constipation, diarrhea, Bristol Stool Scale, treatment, antispasmodics, disorder of gut–brain interaction, FODMAP

## Abstract

Irritable bowel syndrome (IBS) is a highly prevalent and debilitating disorder of gut–brain interaction (DGBI) affecting millions globally. It imposes a significant burden on healthcare systems and is a leading cause of workplace absenteeism. IBS is classified into several subtypes based on predominant presenting symptoms, including IBS with constipation (IBS-C) and IBS with diarrhea (IBS-D), with each requiring targeted approaches to treatment. Some treatments, such as psychotherapy, dietary intervention, and medications like tricyclic antidepressants, are nonspecific and recommended for managing IBS symptoms across all subtypes. In contrast, therapies like secretagogues for IBS-C and eluxadoline or rifaximin for IBS-D are subtype-specific. However, many IBS treatments carry conditional recommendations and are based on low-certainty evidence, emphasizing the need for further research to expand the available treatment options. This review compares the latest IBS management guidelines from the American Gastroenterological Association (AGA), American College of Gastroenterology (ACG), British Society of Gastroenterology (BSG), and European Society for Neurogastroenterology and Motility (ESNM). Pharmacologic and non-pharmacologic therapies, including established and emerging interventions, will be explored to provide a comprehensive guide to management.

## 1. Introduction

The manifestations of gastrointestinal disease are diverse, encompassing conditions that span from mildly to severely debilitating. Irritable bowel syndrome (IBS) is a disorder of gut–brain interaction (DGBI) that is characterized by alterations in bowel habits and is typically accompanied by abdominal pain or discomfort. Although the etiology of IBS is not fully understood, it is believed to involve an interaction between gut–brain axis dysfunction, microbiome disturbances, and visceral hypersensitivity [1,2,3]. The formal diagnosis of IBS is made using the Rome IV criteria, which categorizes gastrointestinal disorders based on the presence of specific chronic, recurrent symptoms without a clear biochemical or structural cause [4]. The Rome IV criteria, introduced in 2016, reclassified IBS from a functional gastrointestinal disorder (FGD) to a disorder of gut–brain interaction (DGBI), reflecting the evolving understanding of its pathophysiology and aiming to reduce stigma. IBS is further classified into subtypes based on the predominance of symptoms: constipation-predominant (IBS-C), diarrhea-predominant (IBS-D), mixed bowel habits (IBS-M), and unclassified (IBS-U) [5].

IBS prevalence is influenced by demographic factors, with women more commonly affected than men and higher rates observed in younger adults compared to those over 40 years of age [6,7,8]. Psychological disorders, such as anxiety and depression, are strongly associated with IBS, and while socioeconomic status (SES) is also considered a risk factor, findings are mixed, with both higher and lower SES linked to increased risk in different studies [9,10,11,12]. A meta-analysis conducted in 2011 estimated the global prevalence of IBS to be approximately 11.2%, but more recent systematic reviews utilizing the Rome IV criteria suggest a lower, though still significant, prevalence of 3.8% [13,14]. Despite these findings, IBS remains a chronic condition that is incapacitating for many and imposes a significant burden on the healthcare system. In the United States, IBS is associated with increased healthcare costs, accounting for 25–50% of referrals to gastroenterologists, and is the second-leading cause of work absenteeism [15,16]. As such, it is imperative for healthcare providers to develop an effective therapeutic alliance with their patients to foster a trusting relationship that encourages open communication. Since the presentation of IBS is highly variable, and the mainstay of treatment is alleviating symptoms rather than curing a pathophysiologic process, care should be individualized to address the specific IBS subtype and symptom profile of each patient. We aim to evaluate the current strategies in the management of IBS, focusing on both pharmacologic and non-pharmacologic approaches, and how they are utilized to treat the various subtypes of this disease.

## 2. Methods

We conducted a literature search for articles published from 1967 to 2024 that focused on the treatment and management of IBS. The primary database used for this search was MEDLINE (PubMed, National Library of Medicine, Bethesda, MD, USA). The search keywords and phrases included ‘disorder of gut–brain interaction’, ‘irritable bowel syndrome’, ‘IBS,’ ‘pharmacologic’, ‘non-pharmacologic’, ‘treatment’, ‘management’, and ‘therapy’. Articles were selected based on methodology, such as study design (e.g., randomized clinical trials, systematic reviews) and the sample size of the study population when applicable. The quality of research was also assessed by evaluating the date and original journal of publication. To ensure that the review reflects the most recent evidence and insights, peer-reviewed articles published in high-impact journals within the past 20 years were prioritized.

## 3. General IBS Treatment Recommendations

IBS is a gastrointestinal disorder without an agreed upon pathophysiologic cause. In the absence of alarm symptoms such as rectal bleeding, unintentional weight loss, or a family history of colon cancer, IBS should be diagnosed with limited testing, relying primarily on clinical history and the Rome IV criteria. The Rome IV criteria for IBS include recurrent abdominal pain occurring at least 1 day per week over the past 3 months, accompanied by at least two of the following: pain that improves or worsens with defecation, a change in stool frequency, or a change in stool appearance. Nearly 75% of patients continue to meet the diagnostic criteria for IBS at the 5-year mark; however, depending on the subtype and severity of the patient’s IBS, as well as treatment modality, long-term treatment has shown improvement of symptoms of up to 50% [17,18].

### 3.1. Non-Pharmacologic Treatments for All IBS Subtypes

The American Gastroenterological Association (AGA), American College of Gastroenterology (ACG), and British Society of Gastroenterology (BSG) have developed general management guidelines applicable to all forms of IBS, and the European Society for Neurogastroenterology and Motility (ESNM) has provided specific recommendations for management of each subtype (Table 1) [19,20,21]. The first-line interventions described within these guidelines primarily consist of comprehensive patient education in conjunction with lifestyle changes (e.g., exercise, stress-reduction, and diet modification). As previously discussed, establishing a strong physician–patient relationship is crucial for addressing concerns and setting realistic expectations for treatment. Physicians should reassure their patients that they are not alone in managing IBS and acknowledge the anxiety this chronic illness may cause. Patients should be encouraged to openly discuss their fears and concerns, as doing so promotes a supportive environment and can help alleviate anxiety [22]. Furthermore, patients should be guided to maintain a realistic outlook on their condition.

#### 3.1.1. Exercise

Exercise has been suggested to reduce the severity of IBS symptoms [23,24]. In one randomized clinical trial (RCT), participants were assigned to a physical activity group, in which they were instructed to increase their exercise levels, and a control group, in which participants maintained their usual lifestyle [25]. Among those in the physical activity group, 43% experienced clinical improvement after completing 20 to 60 min of exercise at least three days per week over a 12-week period. In contrast, only 8% of participants in the exercise group reported worsening symptoms, compared to 26% showing improvement and 23% reporting worsening symptoms in the control group. A systematic review of 14 RCTs involving a total of 683 IBS patients found that physical activity interventions, such as yoga, Tai Ji, and aerobic exercise, significantly improved gastrointestinal symptoms [26]. Another systematic review also had similar conclusions of physical activity possibly improving IBS symptoms [27]. These findings suggest exercise as a feasible treatment option for IBS, though some of the studies were limited by a risk for bias.

However, one potential complication in using exercise as a treatment modality for IBS is low adherence. The cross-sectional observational BE-FIT-IBD study found that, among 219 patients with inflammatory bowel disease (IBD), 42.9% were physically inactive, and only 4.1% participated in health-enhancing physical activity levels [28]. Common barriers to physical activity in these patients included fear of disease flare-ups and concerns that exercise might worsen their condition following diagnosis. If these findings are applicable to IBS, a condition that also presents with lower gastrointestinal symptoms, this underscores the need for providers to actively encourage physical activity as part of IBS management.

#### 3.1.2. Brain–Gut Psychotherapy

Stress reduction plays a significant role in managing IBS symptoms, with brain–gut psychotherapies (BGPs) such as cognitive behavioral therapy for gastrointestinal illness (CBT-GI) and gut-directed hypnotherapy (GDH) emerging as approaches to treatment [29]. Designed to address the pathophysiology associated with gut–brain dysregulation, BGPs serve a dual purpose for IBS patients: first, they help alleviate IBS symptoms closely linked to stress; second, they address anxiety, a common comorbidity [30]. As stress is thought to be associated with many of the symptoms that patients with IBS experience, BGP is a symptom reduction strategy [31]. One meta-analysis found that the benefit of pooled psychological interventions resulted in a number needed to treat (NNT) of two for IBS [32]. Another meta-analysis of 42 RCTs comparing behavioral therapies found that these interventions improved abdominal pain in IBS compared to control groups; however, no intervention was significantly superior to others [33]. Since patients seeking care are often those most anxious about their symptoms, BGP offers a low-risk, long-term alternative to pharmacologic treatments, making it an effective option for many IBS patients.

CBT-GI is the most extensively studied form of BGP and focuses on altering behaviors and thoughts that worsen IBS symptoms [34]. Key techniques include relaxation exercises, cognitive reframing to reduce distressing thoughts, and behavioral experiments to decrease fear and avoidance. Successful CBT-GI can enhance acceptance of the diagnosis, reduce pain perception, and improve psychological flexibility. The AGA has identified over 30 RCTs supporting the use of CBT-GI for IBS in various delivery formats, including individual, group, web-based, and self-administered options. For example, one prospective study found that IBS patients receiving home-based or clinic-based CBT demonstrated significant and sustained improvement in GI symptoms compared to those receiving only education [35]. These benefits notably persisted 12 months later during re-evaluation in a follow-up study [36]. A systematic review and meta-analysis of internet-based CBT-GI demonstrated medium-to-large effects on symptom severity and quality of life [37].

GDH combines traditional hypnotic techniques with targeted suggestions to modulate visceral hypersensitivity, improve gut function, and relieve IBS symptoms [38]. RCTs have primarily assessed subjective symptom relief and explored GDH modalities, such as individual versus group therapy and varying hypnotherapy techniques (e.g., gut-directed hypnotherapy vs. Ericksonian hypnotherapy) [39]. While some studies using validated tools like the IBS Severity Scoring System (IBS-SSS) have shown statistically significant reductions in symptom severity, others have found no difference compared to control groups [40]. Quality-of-life improvements reported by patients also varied widely across studies, with inconsistencies in results attributed to differences in intervention timelines and methodologies. One RCT comparing gut-directed hypnotherapy to supportive treatment found no difference between groups in objective physiological measures, such as gastric emptying time, small bowel transit time, and colonic transit time, after 12 weeks of treatment [41]. CBT-GI and GDH are two distinct but effective non-pharmacologic options for IBS management, and the choice between them can be guided by patient preference and characteristics.

#### 3.1.3. Dietary Modification

Dietary modification has been shown to provide some benefits for managing IBS, though its implementation can be challenging in practice. Before any dietary restrictions are introduced, it is essential to rule out disordered eating. More than 80% of individuals with IBS associate their symptoms with food consumption [42]. Many of these patients will have trigger foods, which are specific meals or ingredients that exacerbate their symptoms. In such cases, the best practice is to encourage patients to avoid these triggers when feasible. However, food avoidance may not be an option for individuals with food insecurity or comorbid dietary restrictions. A registered dietitian can be valuable in providing education and tailored dietary guidance. For patients considering dietary restrictions, it is important to have a discussion regarding the risks and benefits of specific diets.

Two dietary changes, increased soluble fiber intake and the low-FODMAP diet (LFD), have demonstrated efficacy in many IBS patients. Soluble fiber, which absorbs water into the stool and slows digestion, has been strongly recommended by the ACG and BSG for IBS. A systematic review of over 900 patients found that soluble fiber significantly improved IBS symptoms, with the relative risk of remaining symptomatic at 0.86 and a number needed to treat (NNT) of 10 [43]. Insoluble fiber, which adds bulk to the stool to promote regular bowel movements, did not demonstrate significant improvement of symptoms. Soluble fiber sources include psyllium, oat bran, and the flesh of fruits and vegetables, while insoluble fiber is found in wheat bran, whole grains, and seeds. In one RCT of 275 IBS patients, psyllium demonstrated significant symptom relief when compared to a placebo [44]. Symptom improvement from increased soluble fiber intake has been observed in all subtypes of IBS, with the greatest benefit in patients with IBS-C [45].

The low-FODMAP diet (LFD) is one of the most researched dietary interventions for IBS [46]. The diet involves restricting foods high in fermentable oligosaccharides, disaccharides, monosaccharides, and polyols (FODMAPs). These foods include certain fruits (e.g., pears, apples), vegetables (e.g., garlic, onion), dairy products (e.g., milk, soft cheeses), and legumes. It is theorized that these foods increase the delivery of substrates and water to the gut, causing distention of the intestinal lumen and thus triggering pain in IBS patients. LFD is implemented in three phases: restriction, reintroduction, and personalization [47]. In the initial restriction phase, FODMAPs are reduced for 2–6 weeks to assess for symptom improvement. If improvement occurs, the plan is continued, and if there is no improvement, the diet is considered ineffective and stopped. For responders, the next phase involves the gradual reintroduction of FODMAPs over 6–10 weeks, typically one food at a time, allowing for adjustments based on how the patient reacts to these foods. Finally, a personalized diet is developed that the patient can liberalize at their discretion. The LFD has proven to be most effective for patients with IBS-D, and while there is mixed evidence regarding its overall efficacy in other IBS subtypes, systematic reviews and meta-analyses suggest that it offers global symptom relief when compared to standard dietary advice alone [48,49,50]. Therefore, it remains a valuable option, especially for patients seeking non-pharmacologic interventions, and should be considered in collaboration with a GI-trained registered dietitian (GI-RDN).

### 3.2. Pharmacologic Treatments for All IBS Subtypes

#### 3.2.1. Antispasmodics

Antispasmodics are one pharmacologic therapy suggested by the AGA and BSG for managing IBS, though this is not with a strong recommendation. The ESNM weakly recommends the use of antispasmodics in IBS-D but has not recommended them for IBS-C. These medications are thought to ease abdominal pain and cramping by reducing smooth muscle contractions in the gastrointestinal tract [51]. Pharmacologic antispasmodics available in the US include dicyclomine, hyoscyamine, and hyoscine. A Cochrane Review showed significant improvement in IBS symptoms with antispasmodic use, but it was considered low-quality evidence due to a high risk of bias [52]. Conversely, the ACG does not recommend antispasmodics, citing that many studies are of poor quality and outdated [53,54,55].

#### 3.2.2. Peppermint Oil

Peppermint oil, a homeopathic remedy, has demonstrated considerable effectiveness in symptom relief. Its effectiveness is thought to stem from its antispasmodic and anti-inflammatory properties [56]. Both the ACG and AGA conditionally recommend the use of peppermint oil as a substitute for pharmacologic antispasmodics. The BSG weakly recommends peppermint oil for all IBS subtypes and the ESNM weakly recommends it for IBS-D. Multiple RCTs showed significant improvement in IBS symptoms over a placebo, with minimal, well-tolerated adverse effects similar to those of a placebo [57,58]. Another RCT showed clinically significant improvement of symptoms with the IBS-SSS but not statistical significance [59]. A recent meta-analysis found that peppermint oil offers substantial benefits, with an NNT of three for the prevention of persistent symptoms of IBS [60].

#### 3.2.3. Antidepressants

Tricyclic antidepressants (TCAs) have also shown promise in improving IBS symptoms, with an NNT of 4.5 [61]. Their benefit is thought to result through the mediation of norepinephrine and dopamine receptors, thereby reducing psychological distress and visceral pain [62]. At higher doses, TCAs may also slow gastrointestinal transit time, decreasing diarrhea symptoms in IBS-D patients [63]. The BSG and ACG strongly recommend TCAs for global symptoms of IBS, supported by moderate quality of evidence. The AGA conditionally recommends TCAs, while the ESNM provides a consensus recommendation specifically for IBS-D.

Selective serotonin reuptake inhibitors (SSRIs) have previously been considered a possible treatment for IBS. However, RCTs have demonstrated limited efficacy with potential worsening of symptoms [64,65,66,67]. One meta-analysis noted that antidepressants were more likely to improve abdominal pain compared to a placebo; however, this was attributed to TCA use rather than SSRIs [68]. Consequently, the AGA and ESNM have issued conditional recommendations against SSRI use, based on low-certainty evidence, while the ACG offers no recommendation for or against their use [69]. SSRIs have a weak recommendation from the BSG as second-line therapy for global symptoms of IBS but advise careful counseling regarding potential side effects.

## 4. Pharmacologic Treatments for IBS-C

IBS-C is the subtype of IBS in which constipation is the predominant symptom. It meets both Rome IV criteria and is characterized by having specific stool types as defined by the Bristol Stool Scale. Patients with IBS-C have more than 25% of bowel movements classified as types 1 or 2 (hard, pellet-like stools), and fewer than 25% classified as types 6 or 7 (mushy or watery stools). Alternatively, patients may self-report most bowel movements as pellet-like or experience constipation, which is sufficient for clinical diagnosis. The AGA, ACG, ESNM, and BSG have all provided recommendations for the treatment of this form of IBS. In addition to the previously described lifestyle modifications, specific pharmacologic treatments are also considered for IBS-C (Table 2).

### 4.1. Osmotic Laxatives

The first medications to consider for IBS-C management are osmotic laxatives, such as polyethylene glycol (PEG). These laxatives are considered highly effective in treating idiopathic constipation, with an NNT of three across four trials [70]. However, studies have found that PEG does not significantly reduce overall IBS symptoms, including abdominal pain [71]. One RCT found that PEG significantly improved stool consistency and straining, and increased spontaneous bowel movement frequency, but did not significantly decrease pain [72]. Despite this, the AGA recommends PEG as a first-line treatment for IBS-C due to its availability, although this recommendation is conditional with a low quality of evidence. The BSG weakly recommends using PEG, and the ESNM recommends using PEG for chronic constipation but has no specific recommendation regarding its use in IBS-C. In contrast, the ACG advises against its use due to the same concerns about a low quality of evidence but acknowledges its benefit as an easily accessible and affordable option to relieve constipation associated with IBS-C.

### 4.2. Chloride Channel Activators

Secretagogues are a mainstay in the pharmacologic treatment of IBS-C. Lubiprostone, a prostaglandin E1 analog, acts on chloride channels in the intestinal epithelium, increasing fluid secretion and accelerating intestinal transit [73]. It is recommended for IBS-C as it has shown significant improvement in overall IBS-C symptoms with an NNT of 12.5 [74]. Reported adverse effects include nausea and diarrhea, but taking the medication with food has been anecdotally shown to decrease nausea [75]. A meta-analysis of nine trials comprising 1468 patients found lubiprostone provided relief of global IBS symptoms for 3 months after initiating treatment, though only abdominal bloating was significantly improved after 3 months [76]. In 2008, it was approved by the US Food and Drug Administration (FDA) for treatment in adult women with IBS-C [77]. The ACG, AGA, BSG, and ESNM all recommend using lubiprostone for IBS-C, though the BSG and AGA specifically recommend it as a second-line treatment.

### 4.3. Guanylate Cyclase-C Agonists

The ACG and BSG provide a strong recommendation for guanylate cyclase-C receptor agonists, such as linaclotide and plecanatide, for the treatment of IBS-C, supported by a high quality of evidence. The AGA and ESNM agree with these recommendations for linaclotide, though the AGA gives a conditional recommendation for plecanatide while the ESNM gives no recommendation. These medications are believed to improve IBS-C symptoms by stimulating intestinal fluid secretion and enhancing peristalsis, while potentially dampening visceral pain receptors [78]. Linaclotide has been studied in multiple phase III clinical trials, with 34% of patients experiencing symptom relief; it had an NNT of six in a meta-analysis of over 3000 subjects [79,80,81]. Comparatively, plecanatide showed a 26% response rate across studies, with this discrepancy in symptom relief being the reasoning for the AGA’s conditional recommendation.

### 4.4. Serotonin 5-HT4 Receptor Agonists

Tegaserod, a selective serotonin 5-HT4 receptor agonist, has also been used for the treatment of IBS-C and works by stimulating intestinal secretion and motility to alleviate constipation [82]. It was initially removed from the market when retrospective analyses suggested its use led to a higher rate of cardiovascular ischemic events. However, it was reintroduced at a lower dose for use by healthy women under 65 years of age with no history of cardiovascular disease after further studies failed to validate this finding [83]. Tegaserod has demonstrated significant improvement in the global relief of IBS-C symptoms, with 52% of patients reporting symptom reduction, though it did not lead to significant improvements in quality of life [84,85]. Due to these mixed outcomes, tegaserod was given a conditional recommendation by both the AGA and ACG. It is considered an effective second-line treatment for IBS-C by the BSG, though it is no longer available outside of the US. Additionally, like other drugs in this category, tegaserod is associated with diarrhea as a common adverse effect due to its mechanism of action.

### 4.5. Sodium Hydrogen Exchanger 3 Inhibitors

Tenapanor is a sodium hydrogen exchanger 3 (NHE3) inhibitor and was FDA-approved in 2019 for the treatment of IBS-C. By inhibiting NHE3, tenapanor reduces intestinal sodium absorption and increases water secretion into the lumen, which softens stool and accelerates intestinal transit time [86]. Although the mechanism by which it alleviates abdominal pain is unclear, it is thought to involve reducing visceral hypersensitivity. Three controlled RCTs, including two phase 3 trials, demonstrated that tenapanor significantly improved stool frequency, abdominal pain, and overall symptom relief in patients with IBS-C compared to a placebo over 12-to-26-week periods [87,88,89]. The BSG strongly recommends tenapanor as a second-line treatment for IBS-C but warns of diarrhea as a common side effect. The AGA conditionally recommends tenapanor, noting that diarrhea frequently led to treatment discontinuation.

## 5. Pharmacologic Treatments for IBS-D

IBS-D is the subtype of IBS in which diarrhea is the predominant symptom. It meets both Rome IV criteria and is characterized by having specific stool types as defined by the Bristol Stool Scale. Patients with IBS-D have more than 25% of bowel movements classified as types 6 or 7, and fewer than 25% classified as types 1 or 2. Alternatively, if patients report stool appearance consistent with diarrhea, that may be sufficient for clinical diagnosis. The pharmacological treatments of IBS-D will be discussed in detail below (Table 3).

### 5.1. Opioid Receptor Agonists

Loperamide, a peripheral mu-opioid receptor agonist, is commonly used for managing IBS-D. It slows GI motility through decreased peristalsis and an anti-secretory effect [90]. Although effective for reducing diarrhea, loperamide has shown limited efficacy in improving other IBS-D symptoms [91]. Pooled analyses of two RCTs evaluating loperamide for IBS-D found significant improvement in stool frequency but no substantial relief of global IBS-D symptoms [92,93]. The BSG and ESNM strongly recommend loperamide as an anti-diarrheal for IBS-D, recognizing its efficacy but cautioning about potential side effects, including abdominal pain, bloating, constipation, and nausea. Due to these side effects, the AGA conditionally recommends loperamide, whereas the ACG has not made a recommendation regarding its use.

### 5.2. Mixed Opioid Receptor Agonists/Antagonists

Eluxadoline is strongly recommended by the ESNM for treating IBS-D patients and weakly recommended by the BSG. Both the ACG and AGA conditionally recommend eluxadoline for IBS-D. Eluxadoline is a mu and kappa opioid receptor agonist and delta receptor antagonist that has been shown to be effective in two phase III clinical trials [94]. It demonstrated a statistically significant reduction in daily abdominal pain, with a greater than 30% decrease on more than half of the evaluation days, and improved stool consistency, achieving a Bristol Stool Type score of less than five on those days. Eluxadoline also demonstrated a clinically significant increase in IBS quality of life measures when assessed using the IBS-QoL questionnaire, a health-related quality of life measure that has been deemed to have validity in detecting changes due to treatment intervention [95]. The most commonly reported side effects of eluxadoline include constipation and nausea. In patients without a gallbladder and in those consuming more than three alcoholic beverages per day, eluxadoline is contraindicated as pancreatitis and sphincter of Oddi spasm have been associated as rare but serious adverse effects [96]. A phase IV study demonstrated significant improvement of IBS symptoms, similarly to previous clinical trials, and had no treatment-related serious adverse events [97].

### 5.3. Non-Absorbed Antibiotics

Rifaximin is another medication used in the treatment of IBS-D, with a strong recommendation from the ACG and ESNM, and weak and conditional recommendations from the BSG and AGA, respectively. Since it is hypothesized that an abnormal gut microbiome contributes to the pathogenesis of IBS, rifaximin, as a non-absorbed antibiotic, can target this microbiome imbalance directly [98]. Both the AGA and ACG recommend a short course of rifaximin for IBS-D. In pooled phase III clinical trials assessing the efficacy of rifaximin compared to a placebo in improving global IBS symptoms (e.g., abdominal pain, stool consistency), over 40% of subjects reported symptom improvement in the month following treatment, a result that was statistically significant compared to a placebo [99]. Two meta-analyses that summarized five RCTs both found that rifaximin was more effective than a placebo in providing relief of global symptoms [100,101].

To assess the drug’s long-term effects, a subsequent phase III trial examined the outcomes of retreatment in patients who initially responded to rifaximin [102]. This trial showed that a greater proportion of patients had a durable response after retreatment and throughout the retreatment process. Although response rates were lower in the retreatment trial compared to the initial trial, significant improvements in abdominal pain and quality of life were observed. Rifaximin is considered both effective and safe, with a recent meta-analysis demonstrating an NNT of 9 and a number needed to harm of 8971 [103].

### 5.4. Serotonin 5-HT3 Receptor Antagonists

Alosetron, a 5-HT3 antagonist, slows intestinal transit and is conditionally recommended by both the AGA and ACG for use in women with severe IBS-D symptoms who have failed conventional therapy. Alosetron use in IBS-D is weakly recommended by the BSG and strongly recommended by the ESNM. Its efficacy has been demonstrated in two meta-analyses, where alosetron improved global IBS symptoms (e.g., stool urgency and frequency) and overall quality of life [104,105]. Alosetron is associated with significant adverse effects, including severe constipation and ischemic colitis. Due to the severity of these effects, individuals using alosetron in the United States were previously required to enroll in governmental Risk Evaluation and Management Strategies (REMSs) programs. This requirement was removed in 2023, as post-market studies have revealed a better safety profile than initially thought [106]. Further investigation into alosetron as a therapy for IBS-D would be beneficial in assessing whether the eligible patient population could be safely broadened.

### 5.5. Bile Acid Sequestrants

Bile acid sequestrants, such as colestipol and colesevelam, have been suggested for treating IBS-D. It is hypothesized that a subset of patients with IBS-D have elevated colonic bile acids, bile acid malabsorption, or both, leading to the increased colonic secretion of fluid and diarrhea [107]. By binding bile acids, these agents reduce colonic exposure, improving stool consistency and decreasing diarrhea [108]. In one study, subjects on colestipol showed significant improvement in IBS symptoms, as measured by the IBS-SSS, though this study was limited by its sample size and open-label design [109]. In two RCTs, colesevelam increased the total delivery of bile acids to stool but produced mixed results regarding stool consistency and other symptoms [110,111]. Although bile acid sequestrants may be beneficial for improving IBS-D symptoms, there are few well-powered trials to support their efficacy. As such, the ACG conditionally recommends against bile acid sequestrants for IBS-D symptoms, while the AGA, BSG, and ESNM have recognized it as a potential treatment option but have not issued formal recommendations.

## 6. Non-Pharmacologic Treatments with Equivocal Evidence

**Figure 1:**
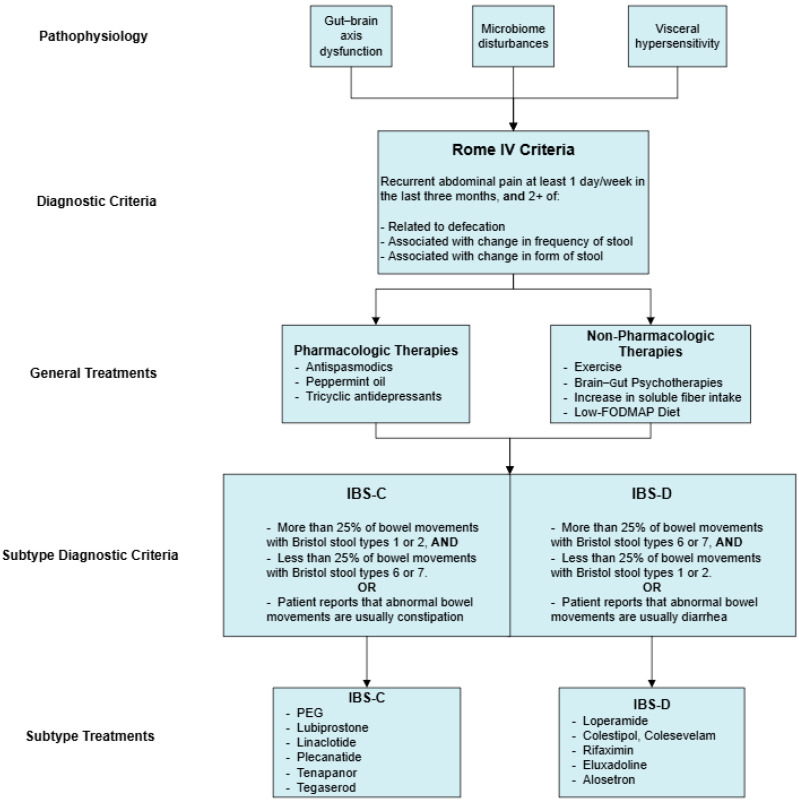
Pharmacologic and non-pharmacologic treatments for IBS and its subtypes.

A summary of the pharmacologic and non-pharmacologic treatments for IBS and its subtypes that have been reviewed can be seen in Figure 1. This following section highlights additional therapies that have been studied in RCTs. These treatments have either equivocal supporting evidence or require further investigation before routine use can be justified.

### 6.1. Biotics

Numerous trials have evaluated the efficacy of prebiotics (non-digestible fibers that promote the growth and activity of beneficial gut bacteria), probiotics (live beneficial microorganisms), and postbiotics (inactivated microorganisms or their metabolites) in alleviating IBS symptom severity, improving quality of life, and reducing psychological symptoms like anxiety and depression. Many of these trials focus on the bacterial genera *Bifidobacterium* and *Lactobacillus*, as well as multi-strain therapies. RCTs have shown positive outcomes, including reductions in depression scores, decreases in IBS-SSS, improved quality of life, and reduced abdominal pain [112,113,114,115,116]. However, there are significant differences across these studies in terms of inclusion criteria, primary endpoints, dosing frequency, and the duration of interventions, which complicates these conclusions. Further data are needed to fully understand the effects of these treatments on the various IBS subtypes, and the mechanistic role of prebiotics and postbiotics. Some trials have specifically evaluated probiotic use in IBS-D and have had positive results [117,118]. Although the evidence surrounding probiotics, prebiotics, and postbiotics is promising, more standardized studies are needed to determine optimal dosing, frequency, timing, and the subtypes of IBS most likely to benefit from these interventions.

### 6.2. Low-Gluten Diet

Aside from the low-FODMAP diet, a low-gluten diet has also been proposed as a treatment option for IBS, though the mechanisms by which gluten affects IBS remain unclear. One RCT found that IBS patients on a gluten-free diet had significantly greater improvements in IBS-SSS, fewer loose stools, and differences in fecal microbiota and metabolite profiles compared to those on a gluten-containing diet [119]. However, another study focusing on non-constipated IBS patients found no significant differences in IBS-SSS between patients following traditional, gluten-free, or low-FODMAP diets, though it noted that the traditional diet was the easiest to adhere to in daily life [120]. A different RCT noted that IBS-D patients on a gluten-containing diet had greater small bowel permeability and higher stool frequency compared to those on a gluten-free diet [121]. These effects were more pronounced in patients who tested positive for HLA-DQ2 and HLA-DQ8 genetic markers, suggesting that the benefits of a gluten-restricted diet may vary based on genotype.

## 7. Conclusions

IBS is a complex disease that impacts millions globally and has a pathophysiology that is not yet fully understood. Its treatment remains focused solely on symptom management as no definitive cure exists. Many theories on IBS etiology have been explored, leading to the development of numerous therapies targeting proposed causes of disease including gut–brain interaction, microbiome balance, stress reduction, and bowel motility. IBS treatments are highly variable, with interventions ranging from lifestyle modifications to dietary adjustments, pharmacologic treatments, and alternative therapies such as hypnotherapy. The inconsistency in IBS presentations further complicates its management, as the subtypes, IBS-C, IBS-D, and IBS-M require tailored approaches with treatment regimens often individualized on a per-patient basis. Healthcare providers should work collaboratively with their patients to develop these personalized management plans, while also taking into consideration the recommendations of medical associations like the ACG, AGA, BSG, and ESNM.

While pharmacologic options such as antispasmodics, secretagogues, and mixed opioid receptor agonists/antagonists have demonstrated efficacy, non-pharmacologic therapies like the low-FODMAP diet and brain–gut psychotherapies should always be considered in treating IBS. Although the medical associations are generally in agreement, the variability in recommendations for certain therapies likely reflects the mixed results of available evidence. Thus, further research is needed to definitively establish the long-term safety and efficacy of these treatments. Additionally, investigations into the underlying mechanisms of IBS, such as in the fields of genetics and the microbiome, could have a profound effect on the direction of treatment and could lead to more targeted, individualized therapies that not only reduce the burden of IBS on the healthcare system but also, more importantly, provide lasting symptom relief and improve the quality of life for IBS patients.

## Figures and Tables

**Table 1:** Pharmacologic and Non-Pharmacologic Treatments for all IBS Subtypes.

		American College of Gastroenterology		American Gastroenterological Association		British Society of Gastroenterology		European Society of Neurogastroenterology and Motility	
Treatment	Class	Recommendation for	Recommendation Against	Recommendation for	Recommendation Against	Recommendation for	Recommendation Against	Recommendation for	Recommendation Against
Low-FODMAP Diet	Lifestyle Modification	Conditional recommendation; very low quality of evidence	—	Best Practice Advice	—	Weak recommendation; very low quality of evidence	—	Strong recommendation; low level of evidence *	—
Increased Soluble Fiber Intake	Lifestyle Modification	Strong recommendation; moderate quality of evidence	—	Best Practice Advice	—	Strong recommendation; moderate quality of evidence	—	—	—
Exercise	Lifestyle Modification	Weak recommendation; very low quality of evidence	—	—	—	Strong recommendation; weak quality of evidence	—	—	—
Cognitive Behavioral Therapy, Gut-Directed Hypnotherapy	Brain–gut Psychotherapy	Conditional recommendation; very low quality of evidence	—	—	—	Strong recommendation; low quality of evidence	—	Strong recommendation; low level of evidence *	—
Dicyclomine, hyoscyamine	Antispasmodics	—	Conditional recommendation; low quality of evidence	Conditional recommendation, low certainty in evidence	—	Weak recommendation; very low quality of evidence	—	Weak recommendation; low level of evidence *	—
Peppermint Oil	Antispasmodics	Conditional recommendation; low quality of evidence	—	Conditional recommendation, low certainty in evidence	—	Weak recommendation; very low quality of evidence	—	Weak recommendation; low level of evidence *	—
Amitriptyline, desipramine	Tricyclic Antidepressants	Strong recommendation; moderate quality of evidence	—	Conditional recommendation; Low certainty in evidence	—	Strong recommendation; moderate quality of evidence	—	Consensus recommendation; unable to assess level of evidence *	—
Fluoxetine, paroxetine	Selective Serotonin Reuptake Inhibitors	—	—	—	Conditional recommendation, low certainty in evidence	Weak recommendation; low quality of evidence	—	—	Conditional recommendation; very low level of evidence *

* = for IBS-D.

**Table 2:** Pharmacologic treatments for IBS-C.

		American College of Gastroenterology		American Gastroenterological Association		British Society of Gastroenterology		European Society of Neurogastroenterology and Motility	
Treatment	Class	Recommendation for	Recommendation Against	Recommendation for	Recommendation Against	Recommendation for	Recommendation Against	Recommendation for	Recommendation Against
Polyethylene Glycol	Osmotic laxatives	—	Conditional recommendation; low quality of evidence	Conditional recommendation; low certainty in evidence	—	Weak recommendation; very low quality of evidence	—	—	—
Lubiprostone	Chloride Channel Activators	Strong recommendation; moderate quality of evidence	—	Conditional recommendation; moderate certainty in evidence	—	Strong recommendation; moderate quality of evidence	—	Strong recommendation; high level of evidence	—
Linaclotide	Guanylate Cyclase-C Agonists	Strong recommendation; high quality of evidence	—	Strong recommendation; high certainty in evidence	—	Strong recommendation; high quality of evidence	—	Strong recommendation; high level of evidence	—
Plecanatide	Guanylate Cyclase-C Agonists	Strong recommendation; high quality of evidence	—	Conditional recommendation; moderate certainty in evidence	—	Strong recommendation; high quality of evidence	—	—	—
Tegaserod	Serotonin 5-HT4 Receptor Agonists	Conditional recommendation; low level of evidence *	—	Conditional recommendation; moderate certainty in evidence **	—	Strong recommendation; moderate quality of evidence	—	—	—
Tenapanor	Sodium Hydrogen Exchanger 3 Inhibitors	—	—	Conditional recommendation; moderate certainty in evidence	—	Strong recommendation; high quality of evidence	—	—	—

* = in women younger than 65 years with ≤1 cardiovascular risk factors; ** = in women younger than 65 years without history of cardiovascular ischemic events.

**Table 3:** Pharmacologic treatments for IBS-D.

		American College of Gastroenterology		American Gastroenterological Association		British Society of Gastroenterology		European Society of Neurogastroenterology and Motility	
Treatment	Class	Recommendation for	Recommendation Against	Recommendation for	Recommendation Against	Recommendation for	Recommendation Against	Recommendation for	Recommendation Against
Loperamide	Opioid Receptor Agonists	—	—	Conditional recommendation; very low certainty in evidence	—	Strong recommendation; very low quality of evidence	—	Strong recommendation; low level of evidence	—
Rifaximin	Non-Absorbed Antibiotics	Strong recommendation; moderate level of evidence	—	Conditional recommendation; moderate certainty in evidence	—	Weak recommendation; moderate quality of evidence	—	Strong recommendation; high level of evidence	—
Eluxadoline	Mixed opioid Receptor Agonists/Antagonists	Conditional recommendation; moderate quality of evidence	—	Conditional recommendation; moderate certainty in evidence *	—	Weak recommendation; moderate quality of evidence	—	Strong recommendation; high level of evidence	—
Alosetron	Serotonin 5-HT3 Receptor Antagonists	Conditional recommendation; low quality of evidence **	—	Conditional recommendation; moderate certainty in evidence ***	—	Weak recommendation; moderate to high quality of evidence	—	Strong recommendation; moderate level of evidence	—
Colestipol, Colesevelam	Bile Acid Sequestrants	—	Conditional recommendation; very low level of evidence	—	—	—	—	—	—

* = contraindicated in patients without a gallbladder or those who drink more than 3 alcoholic beverages per day; ** = in women with severe symptoms who failed conventional therapy; *** = in women with severe symptoms under a risk-management program.

## Data Availability

No new data were created or analyzed in this study. Data sharing is not applicable to this article.
